# International trade regulations take a limited bite out of the shark fin trade

**DOI:** 10.1126/sciadv.adz2821

**Published:** 2025-11-05

**Authors:** Diego Cardeñosa, Elizabeth A. Babcock, Stanley K. Shea, Huarong Zhang, Kevin A. Feldheim, Feng Yang, Stephan W. Gale, Daniel Fernando, Akshay Tanna, Luke Warwick, Demian D. Chapman

**Affiliations:** ^1^ Department of Biological Sciences, Florida International University, North Miami, FL 33181, USA.; ^2^ Rosenstiel School of Marine, Atmospheric and Earth Science, University of Miami, Miami, FL 33149, USA.; ^3^ BLOOM Association, Central, Hong Kong S.A.R., China.; ^4^ The ADM Capital Foundation Ltd, Suite 2405, 9 Queen's Road Central, Hong Kong S.A.R., China.; ^5^ Kadoorie Farm and Botanic Garden, Tai Po, Hong Kong S.A.R., China.; ^6^ Pritzker Laboratory for Molecular Systematics and Evolution, The Field Museum, Chicago, IL 60605, USA.; ^7^ Blue Resources Trust, Colombo 00700, Sri Lanka.; ^8^ Global Marine Program, Wildlife Conservation Society, Bronx, NY 10460, USA.; ^9^ Sharks and Rays Conservation Research Program, Mote Marine Laboratory & Aquarium, Sarasota, FL 34236, USA.

## Abstract

International trade is a major driver of shark overexploitation. In 2013, five threatened shark species were listed on Appendix II of the Convention on International Trade of Endangered Species to regulate global trade and promote recovery. Once listed, any uncertified, unreported export of these species became illegal. Minimal trade was reported from 2015 to 2021, yet fins from four of these species were common in the world’s largest shark fin hub (Hong Kong) throughout this period, indicating substantial and sustained illegal trade. Seventy three of 90 shark fin–exporting nations (81%) have never reported any trade of these species. Mixed stock analysis of a market sample of fins from one listed species revealed six populations of origin but only three were from regions where trade was reported. Broader application of CITES compliance mechanisms is necessary to combat widespread illegal trade of shark fins and realize the conservation potential of these trade regulations.

## Teaser

Survey uncovers widespread illegal shark fin trade despite nearly a decade of international regulation.

## INTRODUCTION

A high proportion of shark species (>37%) are threatened with extinction due to overexploitation, risking the integrity of global marine ecosystems (*1*–*3*). Overexploitation is driven by unmanaged or poorly managed fisheries in most nations, coupled with domestic demand for low-value protein and/or international demand for high-value shark products such as dried fins (*4*–*6*). While the relative importance of domestic and export markets varies by nation, international trade can incentivize the targeted capture or retention of shark bycatch far beyond what is required to satisfy the domestic market (*7*). Thus, regulation of international trade has become an important component of broader shark management efforts (*8*, *9*). The Convention on International Trade in Endangered Species of Wild Fauna and Flora (CITES) Appendix II allows international trade of listed species, provided exporting Parties (i.e., nations/territories) certify that trade involves legally acquired specimens according to national laws, is transparent throughout the supply chain, and is sustainable. The implied CITES Appendix II theory of change is that effective implementation penalizes actors engaged in illegal, opaque, and/or unsustainable international trade that threatens the listed species, thereby reducing a key pressure and contributing to population stabilization and eventual recovery (*10*). This will be most effective in situations where international demand is the primary driver of overexploitation (*10*).

**Fig. 1.**
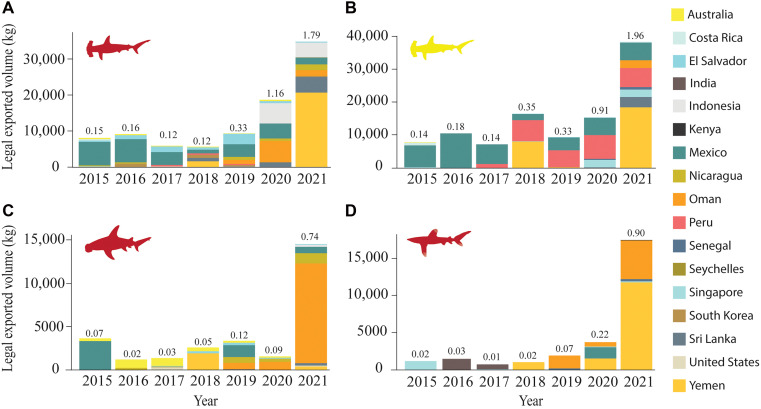
CoP16 species reported trade to Hong Kong by each exporting CITES Party. Bar plot showing the volume of exports to Hong Kong by each trading partner for: (**A**) scalloped hammerhead, (**B**) smooth hammerhead, (**C**) great hammerhead, and (**D**) oceanic whitetip sharks. Yellow and red shark shapes represent vulnerable and critically endangered IUCN Red List categories, respectively, for each species. The color-coding bars depicts the contribution of each exporting nation that reported trading CITES-listed sharks with this hub between 2015 and 2021 (*N* = 18). The numbers above the bars show, for each year, the share of total fin imports into Hong Kong that came from legal trade. Porbeagle sharks are excluded because there were no CITES-reported trade records for this species during the study period.

In 2013, nations party to CITES voted at the 16th Conference of the Parties (CoP16) to list five large, widely distributed shark species on Appendix II (*11*): scalloped hammerhead (*Sphyrna lewini*), smooth hammerhead (*S. zygaena*), great hammerhead (*S. mokarran*), oceanic whitetip (*Carcharhinus longimanus*), and porbeagle (*Lamna nasus*). Unlike the three sharks previously listed on this Appendix [i.e., whale shark (*Rhincodon typus*), great white shark (*Carcharodon carcharias*), and basking shark (*Cetorhinus maximus*)], the CoP16 listings enabled regulation of overexploited species important in the international shark meat or dried fin trade for the first time (*8*, *10*). These listings were subject to an 18-month delay to allow Parties to implement them and entered into force in November 2014. Certification of exports derived from CoP16-listed shark species posed an immediate obstacle to trade because of the threatened status of these sharks (*12*), limited species-specific reporting for the vast majority of nations (*4*), and regional agreements banning retention and all trade of some of these species in some regions, including all three CoP16 hammerhead species by Recommendation 10-08 of the International Commission for the Conservation of Atlantic Tunas (ICCAT), and oceanic whitetip sharks in all tuna Regional Fisheries Management Organizations [tRFMOs; (*2*, *9*, *13*, *14*)]. As is common for species newly listed on CITES Appendix II, nominal trade of these species (Fig. 1) was reported to CITES immediately after entry into force (2015) and 2 years later (2016–2017) their valuable fins remained common in the world’s largest dried shark fin trade hub, Hong Kong Special Administrative Region of the People’s Republic of China, hereafter “Hong Kong” (*8*). This reporting-trade discrepancy indicates that there was an initial “business-as-usual” approach to fishing and trade of these species, with widespread noncompliance with CITES reporting, which is hereafter referred to as “illegal trade” (*8*).

More recent analyses have revealed ongoing discrepancies between relatively large and widespread catches of CITES-listed sharks reported by nations to the United Nations Food and Agriculture Organization (FAO) and/or RFMOs and limited CITES trade records (*15*). These mismatches could be the result of a variety of factors including underreporting of exports and/or catches in Areas Beyond National Jurisdiction (ABNJ) or by fleets operating in foreign jurisdictions (all constituting illegal trade), reporting unit inconsistencies, or ambiguities in reporting requirements between jurisdictions (*15*). While these catch-CITES reporting mismatches could be indicative of illegal trade, another possibility is that products from these CITES-listed sharks largely remain in domestic supply chains, which do not require CITES reporting, rather than entering international markets in substantial quantities. A comparison between CITES reporting and the species composition of international market centers is required to determine whether catch-trade mismatches reflect domestic retention, reporting problems, or ongoing illegal trade (i.e., unreported and uncertified exports).

Here, we address this issue by comparing the occurrence of CoP16 shark species in an established survey of the world’s largest shark fin market (Hong Kong) with contemporary CITES-reported trade from 2015 to 2021. We found minimal CITES reporting did not reflect robust and stable market occurrence of four of the five species over this period, a clear indicator of ongoing illegal trade. We assessed the geographic scope of this illegal shark fin trade in two ways: First, we quantified how many nations are exporting unidentified fins to Hong Kong yet are not reporting any CoP16 species to CITES, including additional factors that indicate potential involvement in illegal activity. This was supplemented by genetically tracing the fins from the most traded CoP16 species (scalloped hammerhead, *S. lewini*) to their region of origin, revealing many discrepancies between where reported trade is occurring and the geographic origins of market derived fins. Relatively few shark fin trading nations are reporting to CITES and there is direct evidence of scalloped hammerheads in the market from regions where there is little or no CITES reporting occurring. We conclude there is widespread illegal trade of fins from four of the five CoP16-listed shark species occurring nearly a decade after these historic listings took force.

## RESULTS

**Fig. 2.**
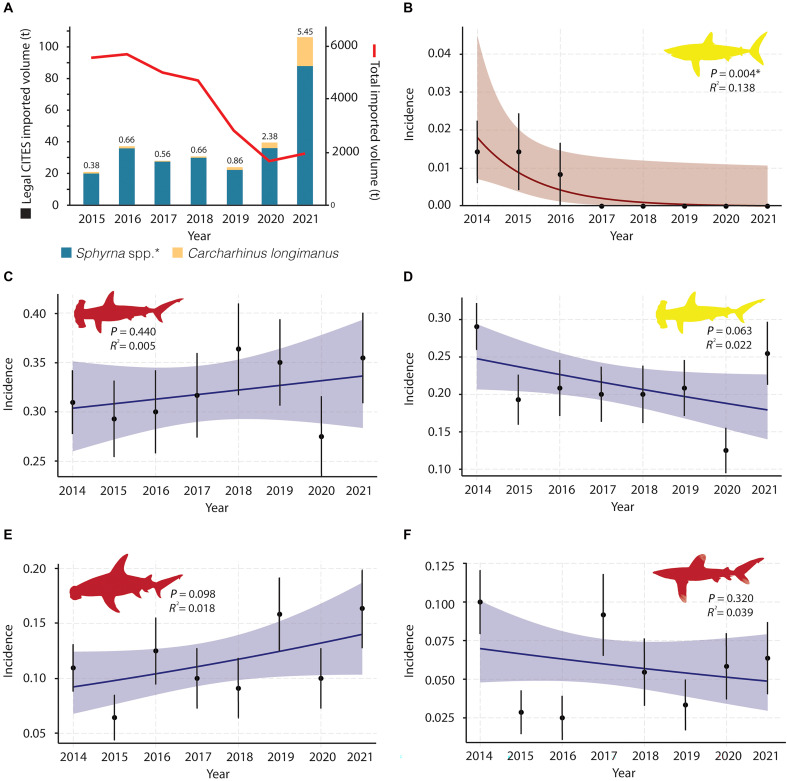
Trends in trade and incidence of CoP16 CITES–listed species in the Hong Kong market. (**A**) Plot showing the legal reported volume from CoP16 species (bars) and the decline in total imported volume of shark fins into Hong Kong (red trend line). Numbers above bars represent the percentage (%) of the legal CITES imported volume of the total imported volume into Hong Kong. Incidence trends of the (**B**) porbeagle, (**C**) scalloped hammerhead, (**D**) smooth hammerhead, (**E**) great hammerhead, and (**F**) oceanic whitetip sharks. Red trends represent significant changes from 2014 to 2021, and blue trends represent nonsignificant changes in that same period. Shaded areas indicate 95% confidence intervals. Yellow and red shark shapes represent vulnerable and critically endangered IUCN Red List categories, respectively, for each species. Porbeagle sharks are excluded from plot (A) because there were no CITES-reported trade records for this species during the study period.

Hong Kong’s total shark fin imports, which are reported without species-level resolution, declined from approximately 6000 to 2000 tons between 2015 and 2021 (Fig. 2A). Despite this decline, Hong Kong remained the largest legal importer of CoP16-listed species according to the CITES Trade Database, accounting for over 67% of reported global imports across this period (https://trade.cites.org/; data S1). However, reported imports (i.e., legal trade) of CoP16 species comprised only 0.4 to 0.9% of Hong Kong’s total fin imports from 2015 to 2019 (Fig. 2A). In 2020 and 2021, total fin imports to Hong Kong reached their lowest recorded volumes, but the proportion of CoP16 species increased from 2.4 to 5.5% due to unusually high CoP16 species exports reported by Yemen and Oman (Fig. 1). Thus, if CITES reporting is accurate, these species should be very rare in the fin market, with an increase in oceanic whitetips, and scalloped, smooth, and great hammerheads from the Indian Ocean population in 2020 to 2021 (Fig. 1).

The species composition of the Hong Kong shark fin market was assessed through DNA analyses of fin trimmings—small pieces of tissue excised from recently imported (within 1 year) whole fins. Trimmings are sold by retail and wholesale vendors for low prices and used in household cooking. A total of 16,222 fin trimmings randomly collected from retail or wholesale vendors between 2014 and 2021 were DNA barcoded to the species level. For this analysis, a sampling event was defined as a visit to a single vendor, during which 20 trimmings were analyzed. In 2014, biweekly sampling produced up to 20 vendor visits per month, while from 2015 onward monthly sampling produced up to 10 vendor visits per month. The incidence of each CoP16 species, measured as the percentage of sampling events in which at least one of the 20 trimmings was identified as that species, remained high (5 to 36% annually, depending on species) and stable across the study period for four of the five CoP16-listed sharks (Fig. 2, B to F).

**Fig. 3.**
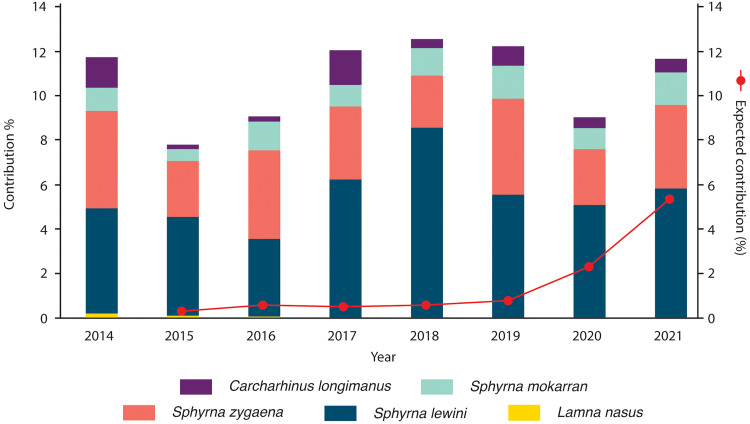
CoP16 species contribution to the Hong Kong market and expected contribution of all CoP16 species based on CITES Trade Database records. Stacked contributions were calculated by dividing the total number of samples identified as each CoP16-listed species by the total number of samples identified in each year. The estimated contribution (red trend line) was calculated by dividing the total volume of all CoP16-listed species imported to Hong Kong, as reported to CITES, by the total import volume reported by Hong Kong’s Agriculture, Fisheries, and Conservation Department.

We also assessed the proportional contribution of each CoP16-listed species to all fin trimmings sampled, which also exhibited stability (Fig. 3). The only exception was the porbeagle shark, which disappeared from our surveys after 2015 (Figs. 2B and 3), consistent with an absence of porbeagle fin exports reported to CITES after that year. In contrast, for the three hammerhead species and oceanic whitetip shark, market presence and CITES reporting diverged substantially. Hammerheads were consistently among the top five most common species in the market [after blue (*Prionace glauca*), silky (*Carcharhinus falciformis*), and blacktip complex (*C. limbatus* and others)], representing 8 to 12% of all fin trimmings annually (Fig. 3), while CITES-reported imports of hammerheads into Hong Kong were typically <1% of all fin imports in the same years (Fig. 1). CITES records of oceanic whitetip imports into Hong Kong were minimal (30.6 tons in total from 2015–2021; ~0.01% of all fin imports; Fig. 1). In contrast, oceanic whitetip fins were common and stable in the Hong Kong market, appearing in 5.7% of annual sampling events on average, with no evidence of decline (Fig. 2F). Their proportional contribution to sampled trimmings (0.7%) was approximately 70 times higher than their reported share of total imports (0.01%; Figs. 1F and 3).

**Fig. 4.**
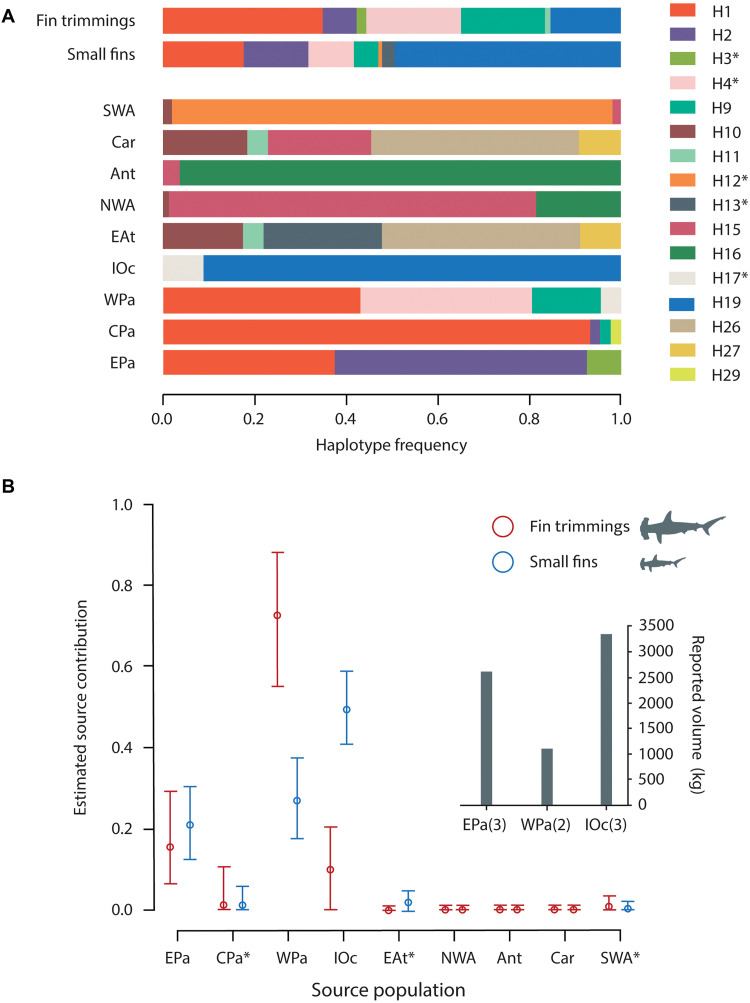
DNA-zip coding of scalloped hammerhead sharks in the Hong Kong market. (**A**) Fields *et al.* (*30*) haplotype frequencies present in the Hong Kong market. Top bars marked as fin trimmings and small fins represent haplotype frequencies from both sampling categories. Lower bars represent haplotype frequencies of each of the potential population sources. SWA, Southwestern Atlantic, Car, Caribbean Sea, Ant, Antilles, NWA, Northwestern Atlantic, Eat, Eastern Atlantic, IOc, Indian Ocean, WPa, Western Pacific, CPa, Central Pacific, EPa, Eastern Pacific. H3*, H4*, H12*, H13*, H17* represent groups of haplotypes that were present exclusively (not shared) in the EPa, WPa, SWA, IOc respectively. (**B**) Estimated contribution of each source population to the shark fin market of Hong Kong with 95% confidence intervals from the MCMC estimation. Small bar plot represents number of CITES Parties per source population that reported scalloped hammerhead shark trade in 2018.

Hong Kong imported dried shark fins from 90 nations between 2014 and 2022, yet only 17 (18.8%) of these reported any trade of CoP16-listed sharks over that period (Table 1). Nearly all of them are range states of one or more of these species and 13 reported catching these species but did not report exporting any of their fins (Table 1). Twenty one of the nonreporting fin trading nations have been implicated as the origin of fin shipments seized at the Hong Kong border for illegally including CITES-listed species (Table 1). Further assessment of the geographic sources of illegal trade was made by DNA zip-coding scalloped hammerhead trimmings and small fins randomly sampled in 2019 to quantify the source contributions of populations living in different geographic regions. A year prior the only nations reporting scalloped hammerhead exports to Hong Kong were Australia and South Korea (Western Pacific population; 1.1 tons combined), Sri Lanka and Yemen (Indian Ocean population; 2.5 tons combined), and Mexico, Peru, and El Salvador (Eastern Pacific and possibly Northwest Atlantic populations; 2.6 tons combined; Fig. 4). DNA zip-coding of scalloped hammerhead fins revealed that the majority originated from the Western Pacific, Eastern Pacific, and Indian Ocean populations, as expected from reported exports (Fig. 4). However, fins also came from the Central Pacific, Eastern Atlantic, and Southwestern Atlantic populations, none of which were associated with any reported scalloped hammerhead exports to Hong Kong, or any other fin importer, from 2015 to 2021 (Fig. 4).

**Table 1.** Summary of nations exporting shark fins to Hong Kong and their involvement with CoP16-listed species. Nations exporting shark fins to Hong Kong (obtained from AFCD under request according to the Code on Access to Information) from 2014 to 2022 in rank order according to mean annual export amount (kg). The number of CoP16 shark species that occur within their waters (out of five total; sourced from www.iucnredlist.org), whether they have reported catching these species to FAO and/or RMFOs [sourced from CITES Secretariat (2024) “Deep diving into shark catch and trade mismatches”] 2014 to 2022, whether shipments exported from the nation have been seized at the Hong Kong border because they lack CITES permits (sourced from AFCD) and whether the country has reported trade of CoP16 species to CITES as legally required (see first paragraph), are also presented. Bolded countries meet the criteria of (1) exporting unidentified fins to Hong Kong, (2) are known be catching CoP16 species in their waters or via their flagged vessels fishing elsewhere and/or have been implicated in illegal trade through Hong Kong’s shark fin seizures, and (3) were not legally trading CoP16 species from 2014 to 2021.

Country of origin	Mean annual imported volume (kg; 2014–2022)	CoP16 range State (*N* species out of 5)	FAO/RFMO reported catches of CoP16 species (2014–2022)	Reported fin shipment seizures in Hong Kong (2015–2022)	Reported CoP16 species to CITES (2014–2022)
**Spain**	725,105.67	5	Yes	No	No
Singapore	605,283	2	No	No	Yes
Senegal	315,602.67	4	No	Yes	Yes
Indonesia	298,621.33	3	No	Yes	Yes
**Taiwan Province of China**	257,287	4	No	No	No
Peru	231,270.89	4	Yes	Yes	Yes
Mexico	144,508.44	4	No	Yes	Yes
Yemen	137,076.33	4	No	Yes	Yes
**United Arab Emirates**	97,553.11	3	No	Yes	No
Argentina	85,395.63	3	No	No	No
United States	83,371.67	5	Yes	No	Yes
Oman	71,103.56	4	Yes	No	Yes
South Africa	68,709	5	No	No	No
**Ecuador**	66,424.86	4	Yes	Yes	No
Sri Lanka	60,168.11	4	Yes	Yes	Yes
**The Mainland of China**	55,632	4	Yes	Yes	No
Japan	50,087.56	4	Yes	No	No
Costa Rica	48,511.67	4	No	Yes	Yes
Uruguay	35,227.57	5	No	No	No
India	34,888.75	4	No	Yes	Yes
**Philippines**	30,270.63	4	No	Yes	No
South Korea	28,031.13	3	No	No	Yes
**Ghana**	27,104.22	4	No	Yes	No
**Brazil**	26,552.22	5	No	Yes	No
Trinidad & Tobago	24,328.5	4	No	No	No
**New Zealand**	23,827.5	3	Yes	No	No
France	22,245.13	5	No	No	No
Togo	19,843.75	3	No	No	No
**Pakistan**	15,342.11	4	No	Yes	No
**Morocco**	14,602.78	5	Yes	Yes	No
El Salvador	14,326.88	3	No	No	Yes
**Guinea**	12,381.38	4	Yes	Yes	No
**Somalia**	12,343.38	4	No	Yes	No
Chile	12,190.78	3	No	No	No
Bangladesh	11,658	3	No	No	No
Namibia	10,725.33	3	No	No	No
**Guyana**	10,206.38	3	No	Yes	No
**Guatemala**	9,935.33	3	No	Yes	No
Australia	9,710.22	5	No	No	Yes
Malaysia	9,642.78	2	No	No	No
**Madagascar**	8,633.11	4	No	Yes	No
Kenya	8,360.89	3	Yes	Yes	Yes
Thailand	8,081.67	3	No	No	No
**Panama**	7,663.38	4	No	Yes	No
**Mauritania**	6,803	4	Yes	No	No
Mozambique	6,719	3	No	No	No
Papua New Guinea	6,587.78	3	No	No	Yes
Tunisia	5,025.13	4	No	No	No
Sierra Leone	4,829.11	3	No	No	No
Colombia	4,629.17	4	No	Yes	Yes
Canada	3,531.67	2	No	No	No
**Nicaragua**	3,481.11	3	No	Yes	No
Angola	3,429.17	3	No	No	No
Mauritius	3,347.8	3	No	No	No
**Venezuela**	3,287.5	3	Yes	Yes	No
Saudi Arabia	3,233.38	4	No	No	No
**Congo**	2,842.33	3	No	Yes	No
Vietnam	2,652	4	No	No	No
Solomon Is	2,462.5	3	No	No	No
**Seychelles**	2,256.22	4	Yes	Yes	No
Germany	2,131.75	2	No	No	No
**Democratic Republic of the Congo**	2,107.25	3	No	Yes	No
**Egypt**	2,002.38	5	No	Yes	No
Cuba	1,607.83	4	No	No	No
Norway	1,357.33	2	No	No	No
Turkey	1,307.86	0	No	No	No
**Fiji**	1,307	3	Yes	No	No
**Iran**	1,106	3	Yes	No	No
Cote D’Ivoire	974	4	No	No	No
Nigeria	880.43	2	No	No	No
Ethiopia	865.83	0	No	No	No
Bahrain	851.89	3	No	No	No
**Liberia**	524.83	4	Yes	No	No
**Tanzania**	441.71	3	No	Yes	No
Aruba	384	3	No	No	No
Belize	307.4	3	No	No	No
Mali	293	0	No	No	No
Macau	226.2	4	No	No	No
Sweden	202	2	No	No	No
Maldives	189.43	3	No	No	No
Uganda	137.4	0	No	No	No
Libya	114	4	No	No	No
Zimbabwe	90	0	No	No	No
Zambia	83.33	0	No	No	No
Kuwait	60.2	3	No	No	No
Cameroon	59.75	4	No	No	No
Vanuatu	58	1	No	No	No
Northern Mariana Islands	46.5	1	No	No	No
Sudan	38.6	3	No	No	No
Dominican Republic	29.4	4	No	No	No

## DISCUSSION

This study reveals major discrepancies between the occurrence of four of five CoP16-listed shark species in the Hong Kong fin market and low levels of trade reported to CITES. If reporting compliance was high and these records were accurate, these species would be rare in the Hong Kong market after listings took force. This expectation was met for the porbeagle shark, where its postlisting disappearance from the market aligned with the lack of reported fin exports. This could have resulted from effective imposition of postlisting retention bans and CITES implementation by a limited number of high-capacity fishing nations that typically caught this species [i.e., United States, Canada, Australia, and New Zealand; (*15*, *16*)]. However, it could also reflect how depleted this species is in some ocean basins (*17*).

In contrast to porbeagles, CITES reporting and market contribution of the other four CoP16-listed species did not align. Three of these are coastal or semi-pelagic hammerhead sharks that are mainly caught in countries where shark fisheries are not sustainably managed (*13*), making it difficult to issue positive CITES nondetriment findings (NDFs), which are formal scientific assessments required under Article IV of CITES to demonstrate that proposed exports are not detrimental to the survival of the species in the wild, and a prerequisite for issuing any export permit. Although CITES records show that hammerhead sharks typically account for less than 1% of all fin imports into Hong Kong in most years (*2*, *18*), our sampling consistently found that these three hammerhead species represented 8 to 12% of all fin trimmings each year and have typically ranked as the fourth and fifth most common species in the market overall (*2*, *5*). The last of the CoP16-listed species, the oceanic whitetip shark, is mainly caught by industrial pelagic fisheries targeting tuna and retention is prohibited by all tRFMOs that govern these fisheries (*19*). CITES reported imports of this species to Hong Kong were minimal (i.e., 0.01% of all fin imports), likely in part because oceanic whitetip shark trade cannot be certified if it contravenes tRFMO retention bans. Contrary to this low reporting, oceanic whitetips were common and stable in the Hong Kong market during 2014 to 2021. While it is theoretically possible that oceanic whitetips and hammerheads could be overrepresented in fin trimmings relative to their presence in imported whole fins, our sampling effort, comprising over 16,000 barcoded trimmings collected over a 7-year period using randomized vendor selection and consistent monthly sampling, minimizes this concern. Our results provide robust evidence of sustained illegal trade of the fins of these species since listings took force in late 2014.

Sustained illegal trade of fins from these species likely involves many shark fishing and trading nations. Over 80% of the nations that exported unidentified shark fins to Hong Kong from 2014 to 2022 did not report any trade of CoP16-listed sharks to CITES. While some of these nations may only be trading fins from other species, it is highly unlikely that very large exporters such as Spain, Taiwan Province, and Mainland China (each exporting >50,000 kg unidentified shark fins to Hong Kong annually) that have large industrial fleets operating all over the world that are known to catch these species (*18*, *20*, *21*) are not also exporting their fins. Moreover, close to one-third of nonreporting nations (e.g., Mainland China, United Arab Emirates, Ecuador, Pakistan, Ghana, Brazil, and Philippines) have been directly implicated in the illegal shark fin trade based on fin seizures in Hong Kong. As a further indicator of the widespread nature of the illegal shark fin trade, we also found discrepancies between where scalloped hammerhead fins originate and where reported trade occurs. While DNA zip-coding data revealed that scalloped hammerhead fins primarily originated in the Western Pacific, Eastern Pacific, and Indian Ocean populations, as expected based on reporting, smaller contributions came from the Central Pacific, Eastern Atlantic, and Southwestern Atlantic. These estimates are based on mixed-stock analysis (MSA), which does not assign individual fins to specific locations but instead compares haplotype frequencies in the “mixed” market sample to those in potential source populations to estimate proportional contributions. Because each source population is characterized by multiple distinctive haplotypes, the effect of potential spatial overlap among populations is minimized (*22*).

The relative contributions of the Western Pacific and Indian Ocean populations to the Hong Kong market in 2019 are also suspicious. In 2018, only two nations fishing the Western Pacific population reported exporting scalloped hammerhead fins to Hong Kong. This region recorded the smallest legal trade (1.1 metric tons) yet accounted for the highest and second-highest contributions to trimmings and small fins, respectively, indicating likely illegal trade (Fig. 4). The Western Pacific population is fished by at least two of the world’s largest shark-fishing nations, Indonesia (no. 1) and Malaysia (no. 9), neither of which reported any exports of scalloped hammerhead fins to Hong Kong in 2018, despite exporting large quantities of unidentified shark fins to this trade hub (*4*, *23*). Indonesia had an export ban on scalloped hammerheads from 2017 to 2019 to comply with CITES regulations. Encouragingly, Indonesia reported exporting 5.6 metric tons of undifferentiated hammerhead fins to Hong Kong for the first time in 2020, followed by 4.1 metric tons of scalloped hammerhead fins in 2021, along with smaller volumes of great and smooth hammerhead fins. These quantities are comparable to other major exporters from the Eastern Pacific (e.g., Mexico) and the Indian Ocean (e.g., Yemen), suggesting that Indonesia was likely involved in illegal hammerhead fin trade from 2015 to 2020, before gradually improving species-specific reporting in 2020 to 2021. This is further supported by three seizures of fin shipments including this species exported from Indonesia in 2018 to 2019 in Hong (www.info.gov.hk/gia/general/202005/27/P2020052700507.htm). The Indian Ocean was also a major source of scalloped hammerhead fins and the highest in the sample of small fins (Fig. 4), even though Sri Lanka, Yemen, and Oman were, and remained until 2021, the only CITES-reporting nations in this region. This species is caught in large volumes along the east African coast and in South Asia, including Pakistan (no. 8 largest shark-catching nation), Iran (no. 18), Madagascar (no. 29), Bangladesh (no. 36), Tanzania (no. 37), which all regularly export unidentified fins to Hong Kong (*4*). Fin shipment seizures with illegal occurrence of CoP16 species have also been recorded from Seychelles, United Arab Emirates, Madagascar, Somalia, and Kenya in the Indian Ocean region.

The CITES reporting-market mismatches at the species and population level and implausibly low levels of reporting by major fin traders all point to systemic noncompliance leading to substantial illegal trade of shark fins. CITES is unique among multilateral environmental agreements in incorporating compliance mechanisms that can be activated to strengthen implementation by Parties (*9*, *24*). Among these is the Review of Significant Trade (RST), which seeks to ensure that reported trade in Appendix II species is conducted legally, sustainably, and in accordance with Article IV of the Convention (*24*). While RST has recently been applied to a limited number of Parties in relation to questions about their reported trade of CoP16-listed sharks (*25*), the persistent mismatches between reported and actual trade in this study highlight that unreported, and therefore illegal, trade is also a distinct and substantial problem. Article XIII of the treaty offers an additional compliance pathway, enabling the Secretariat to assess potential noncompliance and, where appropriate, provide technical assistance and capacity-building support to Parties (*26*). For example, Article XIII has recently been invoked to suspend all shark trade from Ecuador pending assessment and management improvements (*27*).

The persistent mismatch between market data and reported trade for oceanic whitetip and large hammerhead sharks, observed 7 years after their CoP16 listings, suggests that broader and more strategic use of Article XIII to address illegal trade is warranted alongside RST to address issues with reported trade. We suggest that nonreporting nations known to be trading large quantities of unidentified fins to Hong Kong (e.g., Spain, Taiwan Province, and Mainland China) and especially those already implicated in illegal trade (e.g., Mainland China, United Arab Emirates, Pakistan, Ghana, Brazil, and Philippines; Table 1) should be priority targets for Article XIII action. Given the ongoing decline of many commercially important sharks, the strategic and consistent application of existing compliance tools remains essential. While the pace of change within CITES can be gradual, sustained engagement with its compliance mechanisms is critical to ensure that listed species receive the protections anticipated under the Convention. Outside of CITES, stronger port state measures, mandatory species-level customs codes for shark fins, and coordinated market surveillance (e.g., genetic monitoring of trade hubs) could complement Convention-based enforcement. Addressing the continued trade of hammerhead and oceanic whitetip fins will likely require this combined approach, strengthening CITES from both within and the external governance systems that intersect with it.

## MATERIALS AND METHODS

### Hong Kong trade records

CITES Trade Database records (https://trade.cites.org) were accessed to obtain the volume (kg) of reported, and therefore legal, trade of shark fins to Hong Kong from 2015 to the end of 2021 for all species listed during CoP16. Since we only sampled fins during our Hong Kong market surveys (see below), we excluded all other products and only included trade of known quantities (i.e., weight in kg) from wild-caught specimens (source = W), and only for commercial purposes (purpose = T). For example, there were 11 instances where reported trade of *Sphyrna* spp. and one instance from *C. longimanus* involved unknown quantities from six different countries, accounting for a total of 5026.43 “fins” with no reported weight. There were also many discrepancies between the reported volumes by the importer and the exporter. Therefore, for the analysis presented here, the highest reported volume by any of the trade partners was considered. Overall fin import records for Hong Kong were obtained from the Agriculture, Fisheries and Conservation Department (AFCD) upon request, in accordance with the Code on Access to Information, so that the CITES reports of each CoP16 species could be expressed as a percentage of the imported total fin volumes each year across the same period (2015–2021).

### Hong Kong market surveys

As part of an ongoing long-term survey of shark fin retail markets, Hong Kong’s Sheung Wan District was sampled from February 2014 to December 2021 (*2*). Our survey methodology consisted of the sampling of processed shark fin trimmings that are sold in bags containing 10 to ~3000 pieces. Imported shark fins are rapidly processed either in Hong Kong, neighboring mainland China, or other parts of Southeast Asia. The resulting processed fins and trimmings from the latter two locations are then reimported to Hong Kong for sale (*23*). The trimmings, being of low value and perishable, exhibit a quick turnover (≤12 months), evident from how frequently vendors deplete their stock (*8*). We therefore conservatively assumed that trimmings in the retail market provide an index of the species composition of fin imports within the past year (e.g., trimmings present in 2016 likely reflect imports/processing in 2015).

From February 2014 to February 2015, sampling was conducted every 2 weeks. Each vendor in the district was assigned a unique number, and for every sampling event, 75 vendors were randomly selected without replacement from the full list of ~300. A Hong Kong–based resident visited these vendors in the order selected, purchasing two bags of trimmings from each vendor with stock available. If a selected vendor lacked trimmings, the next vendor on the randomized list was visited. Sampling continued until 10 stocked vendors had been sampled, yielding 20 bags per event. From March 2015 onward, sampling was conducted monthly, using the same randomization and replacement procedure. Subsequently, the contents of each bag were counted, and 10 random samples were selected from each bag for genetic identification following the mini-barcoding protocols by Cardeñosa *et al.* (*28*). A parallel survey of small fins found at the same markets (i.e., fin base <10 cm) was conducted through 2019 (*5*). The survey methodology consisted of sampling small, processed shark fins sold in bags of ~0.6 kg. One bag was purchased from five randomly selected vendors. Two bags were purchased in instances where one single bag was estimated to contain fewer than 100 fins. A total of 95 fins were randomly selected from each vendor for genetic analysis following the protocols by Cardeñosa *et al.* (*28*). The species composition data from this survey are not presented here; however, the fins identified as scalloped hammerhead (*n* = 253) were used to conduct DNA zip-coding (i.e., identification of population sources) together with fin trimmings from the same species in that same year (see section below).

### Analysis of incidence of CoP16 species in Hong Kong markets

A generalized linear model (GLM) with a binomial distribution and a logit link function was employed to predict the probability of incidence for species listed in CoP16 from 2015 to 2021, with numerical year as the only predictor variable to test for a trend over time. Here, we define incidence as the presence of CITES-listed species in a trial, where a trial is defined as a visit to a vendor (i.e., two purchased bags of shark fin trimmings). In 2014, when sampling events occurred twice a month, the maximum possible number of incidences for a single species was 20 (i.e., 20 vendor visits or trials). In the rest of the survey, when sampling occurred once a month, the maximum possible number of incidences per species was 10. Models were conducted using the GLM function in R (*29*). Scaled residuals were examined with the R package DHARMa version 0.4.6 (*30*).

### Assessing CITES reporting by nations exporting fins to Hong Kong 2014 to 2022

To provide an overview of nations that could be participating in the illegal shark fin trade we compiled a list of Hong Kong’s shark fin trading partners based on customs data from 2014 to 2022 obtained under request according to the Code on Access to Information. These data include the mean weight (kg) of fins received from each nation by year, but this is not species specific. Each nation was scored as to whether it reported any exports of CoP16-listed fins to Hong Kong over that period using the CITES Trade Database records (https://trade.cites.org). The potential that the nation catches CoP16 in its shark fisheries was assessed by compiling the number of CoP16 shark species that occur within their waters (out of five total; sourced from www.iucnredlist.org) and whether they have reported catching these species to FAO and/or RMFOs (*15*). Direct evidence of each nation’s participation in the illegal shark fin trade was assessed by examining whether shipments exported from the nation have ever been seized at the Hong Kong border because they lack CITES permits (sourced from AFCD). These metrics were used to identify nations that did not report trade of CoP16 species, yet participated in the fin trade, likely catch these species, and/or have been directly implicated as sources of fins seized at entry in Hong Kong because of a lack of CITES permits.

### DNA zip-coding of scalloped hammerhead

**Fig. 5.**
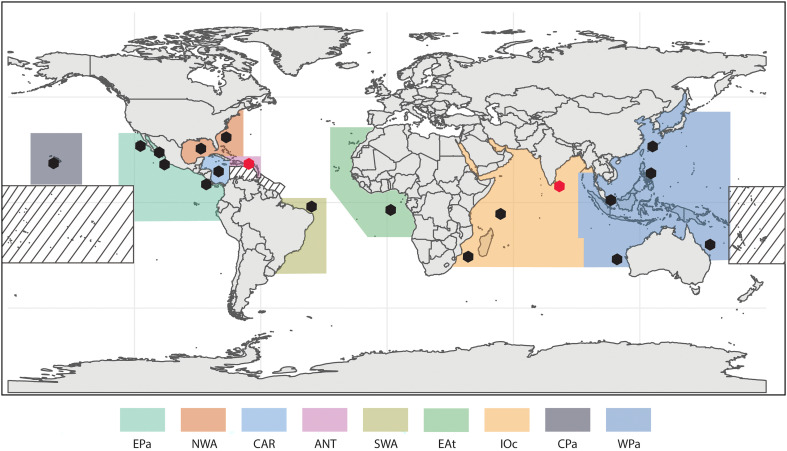
Baseline population genetics data for the scalloped hammerhead shark for the mtCR. Color-shaded areas represent each defined population included in Duncan *et al.* (*31*), Fields *et al.* (*30*) and this study. NWA, Northwestern Atlantic; Car, Caribbean Sea; SWA, Southwestern Atlantic; EAt, Eastern Atlantic; IOc, Indian Ocean; ANT, Antilles; WPa, Western Pacific; CPa, Central Pacific; EPa, Eastern Pacific. Areas with lines represent areas where sampling of scalloped hammerhead shark has not been included in global population genetics studies. Black hexagons represent sampling locations by Duncan *et al.* (*31*), red hexagons represent sampling from this study.

To further assess where illegal shark fins may be coming from, a baseline genetic population structure for the scalloped hammerhead across its geographical range was compiled to assess the relative contribution of these populations to the fin markets of Hong Kong (Fig. 5). Therefore, we used 34 mitochondrial control region (mtCR) haplotypes previously identified across the world that defined eight populations [Southwestern Atlantic (SWA), Caribbean Sea (Car), Northwestern Atlantic (NWA), Eastern Atlantic (EAt), Indian Ocean (IOc), Western Pacific (WPa), Central Pacific (CPa), and Eastern Pacific (EPa); (*31*–*34*)]. All baseline haplotype sequences and frequencies were obtained using the information provided by Fields *et al.* (*31*). We also obtained samples of scalloped hammerheads from previously unsampled regions: Sri Lanka (*N* = 30) and Puerto Rico (*N* = 58). To improve our global baseline, we amplified and sequenced the mtCR from these new samples using the following protocol: genomic DNA was extracted using the DNeasy Qiagen Blood and Tissue Kit following the manufacturer’s instructions and amplified using the primers Pro-L (5′-AGGGRAAGGAGGGTCAAACT-3′) and 12SrRNA (5′-AAGGCTAGGACCAAACCT-3′), following the polymerase chain reaction (PCR) thermal conditions by Duncan *et al.* (*32*). Each PCR reaction comprised 12.5 μl of GoTaq Hot Start Green Master Mix (Promega), 7.5 μl of molecular grade water, 1.5 μl of each primer (10 μM), and 2.0 μl of genomic DNA. PCRs were checked on a 2% agarose gel, and all products were cleaned using ExoSAP-IT (Affymetrix Inc., Santa Clara, CA, USA). Products were sequenced using the BigDye Terminator version 3.1 Cycle Sequencing Kit (Applied Biosystems, Foster City, CA, USA) and run on an ABI 3730 DNA Analyzer. All forward and reverse sequences were checked manually and trimmed using Geneious Pro v3.6.1 (www.geneious.com) to match the length of global haplotypes provided by Fields *et al.* (*31*).

A 2015 study reported a genetically distinct population of scalloped hammerheads in the Arabian Peninsula, characterized by unique haplotypes not found in any other population of the species (*35*). However, none of these haplotypes have been detected in Hong Kong fin market surveys (*31*, *34*). Contrary to Spaet *et al.* (*35*), 28 sequenced samples from the Arabian Peninsula matched Haplotype 19, the most common haplotype in the Indian Ocean. The same was true for all samples from Sri Lanka. Consequently, these samples were grouped with Indian Ocean samples from Duncan *et al.* (*32*) into a single population (IOc) for further analysis. The Puerto Rico samples exhibited two previously described haplotypes from the Western Atlantic but in unusual frequencies. Therefore, an analysis of molecular variance (AMOVA), along with pairwise comparisons with other populations, was performed using Arlequin version 3.5.1 (*36*). Results, presented in tables S1 and S2, indicate that the Puerto Rican samples formed a ninth genetically distinct scalloped hammerhead population, referred to hereafter as the Antilles (Ant; Fig. 5).

Samples identified as scalloped hammerhead in the fin trimmings (*n* = 108) and processed small fins (*n* = 253) collected in 2019 were used to assess the relative contribution of the different global populations described for this species (*31*–*34*). On the basis of fin size, all small fins were assumed to be from juvenile sharks, while shark trimmings were assumed to be from a range of sizes, including adults and juveniles. The global genetic population structure baseline was constructed using a 535 bp informative section of the mtCR (*32*). Smaller amplicons are easier to amplify when the target DNA is degraded and of low quality (*28*). Therefore, because the genomic DNA from our processed market samples was highly degraded (*28*), we designed two internal primers, Slew_mtCR_intR (5′-CGAGCATTAACTATATTGCCAT-3′) and Slew_mtCR_intF (5′-ATGGCAATATAGTTAATGCTCG-3′), that, combined with the original primers (Pro-L and 12SrRNA), amplify two fragments of 388 and 168 bp, respectively. The concatenation of these amplicons yields the 535 bp fragment used in the global baseline. A total of 92 and 142 complete sequences were retrieved from scalloped hammerhead fin trimmings and small fins, respectively. Haplotypes were defined using both internal amplicons (fig. S1) with RStudio (*29*) and the pegas R package (*37*). We used the haplotype frequencies from Fields *et al.* (*31*), along with those from the newly defined Ant population, and the observed frequencies in the fin market (Fig. 4A), to conduct a MSA using the R-package mixstock (*38*). Mixstock was used to estimate the contribution of each source population to the Hong Kong fin markets via a Markov Chain Monte Carlo (MCMC) approach with 100,000 iterations following a burn-in of 50,000. The Gelman and Rubin criterion was used to assess convergence (*39*). All parameters converged based on the Gelman and Rubin criterion (<1.2).
