# Supplementary material for: International trade regulations take a limited bite out of the shark fin trade
Source: Sci Adv. 2025 Nov 5;11(45):eadz2821. doi: 10.1126/sciadv.adz2821 (PMC12588279; doi:10.1126/sciadv.adz2821)
Supplement: Supplementary file 1 — Figs. S1 to S6 Tables S1 and S2 Legends for data S1 to S3 [file sciadv.adz2821_sm.pdf]

Science Advances
AAAS

Supplementary Materials for

International trade regulations take a limited bite out of the shark fin trade

Diego Cardeñosa *et al.*

Corresponding author: Diego Cardeñosa, dcardeno@fiu.edu

Sci. Adv. **11**, eadz2821 (2025)
DOI: 10.1126/sciadv.adz2821

The PDF file includes:

Figs. S1 to S6
Tables S1 and S2
Legends for data S1 to S3

Other Supplementary Material for this manuscript includes the following:

Data S1 to S3

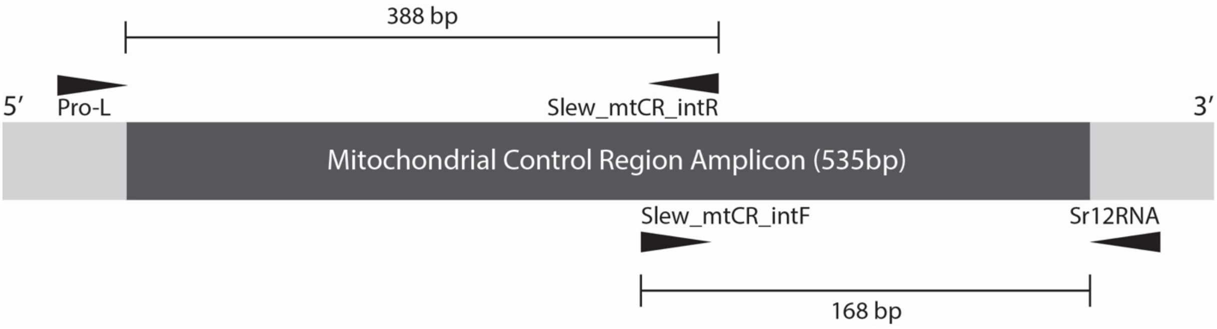

Figure S1. Schematic representation of the relative annealing sites and orientation of each primer (black triangles) and spatial coverage of the resulting amplicons.

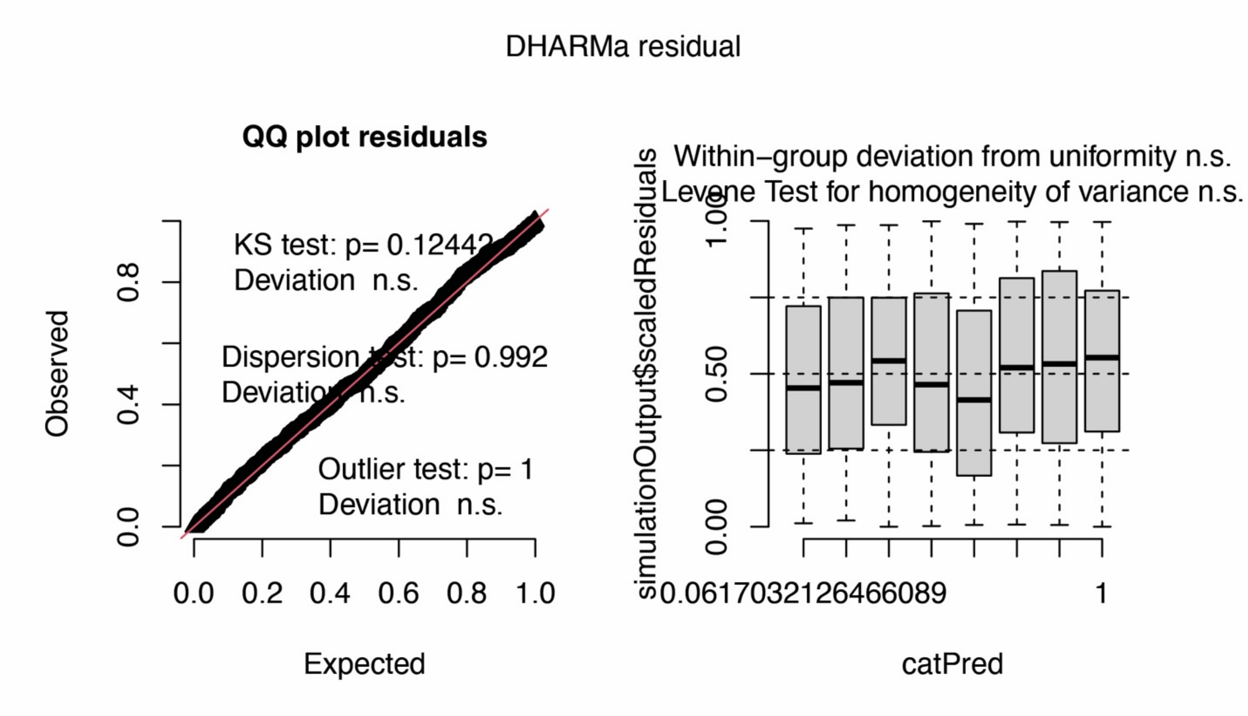

Figure S2. DHARMa residual for the porbeagle model, showing that the binomial model appropriately represented the data.

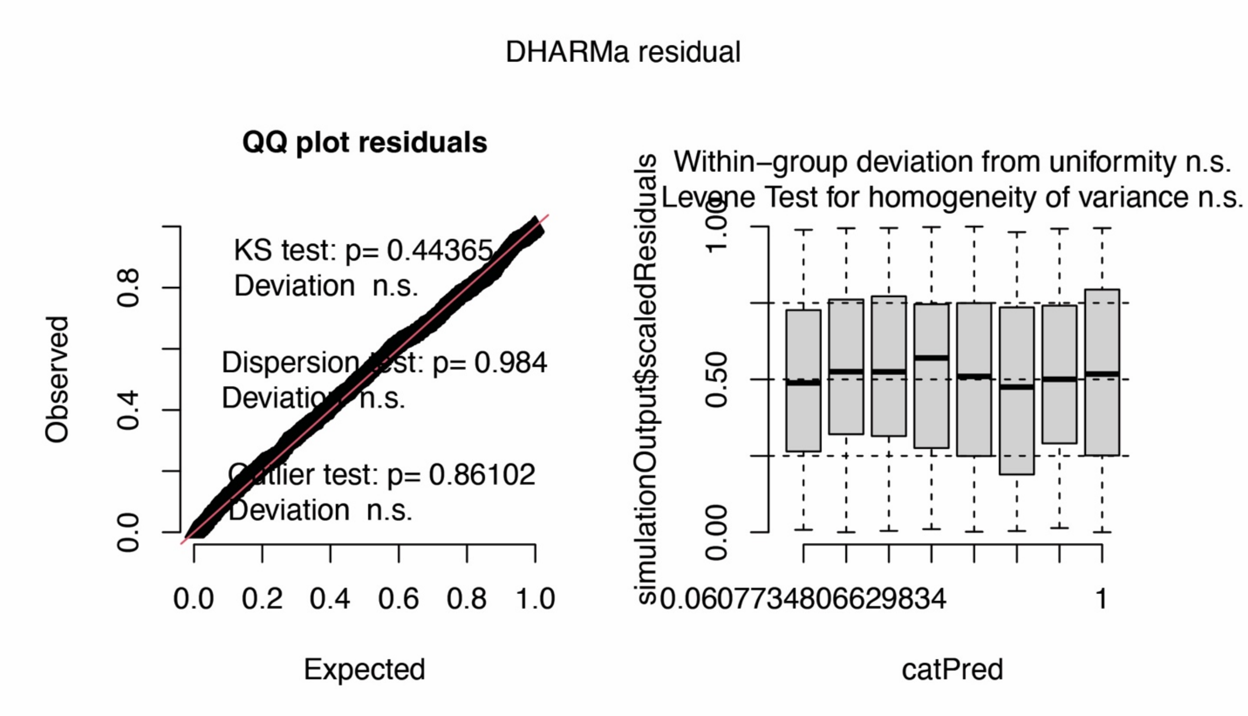

Figure S3. DHARMa residual for the scalloped hammerhead, showing that the binomial model appropriately represented the data.

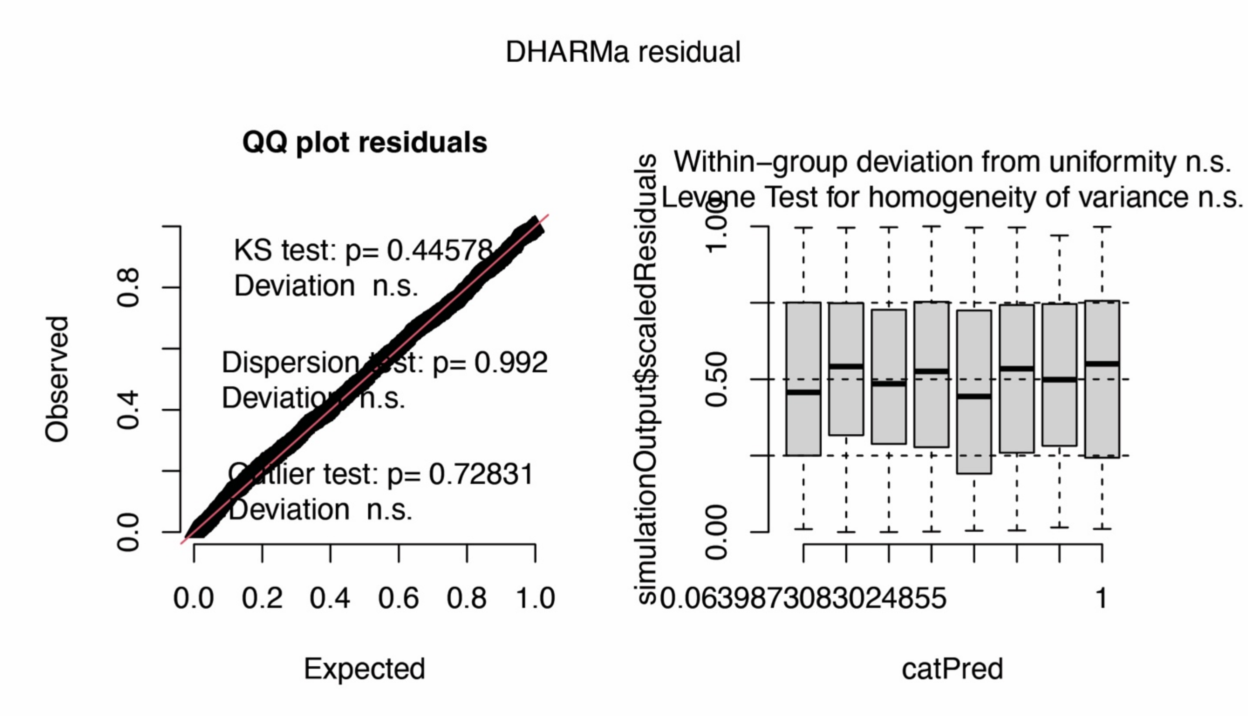

Figure S4. DHARMa residual for the smooth hammerhead, showing that the binomial model appropriately represented the data.

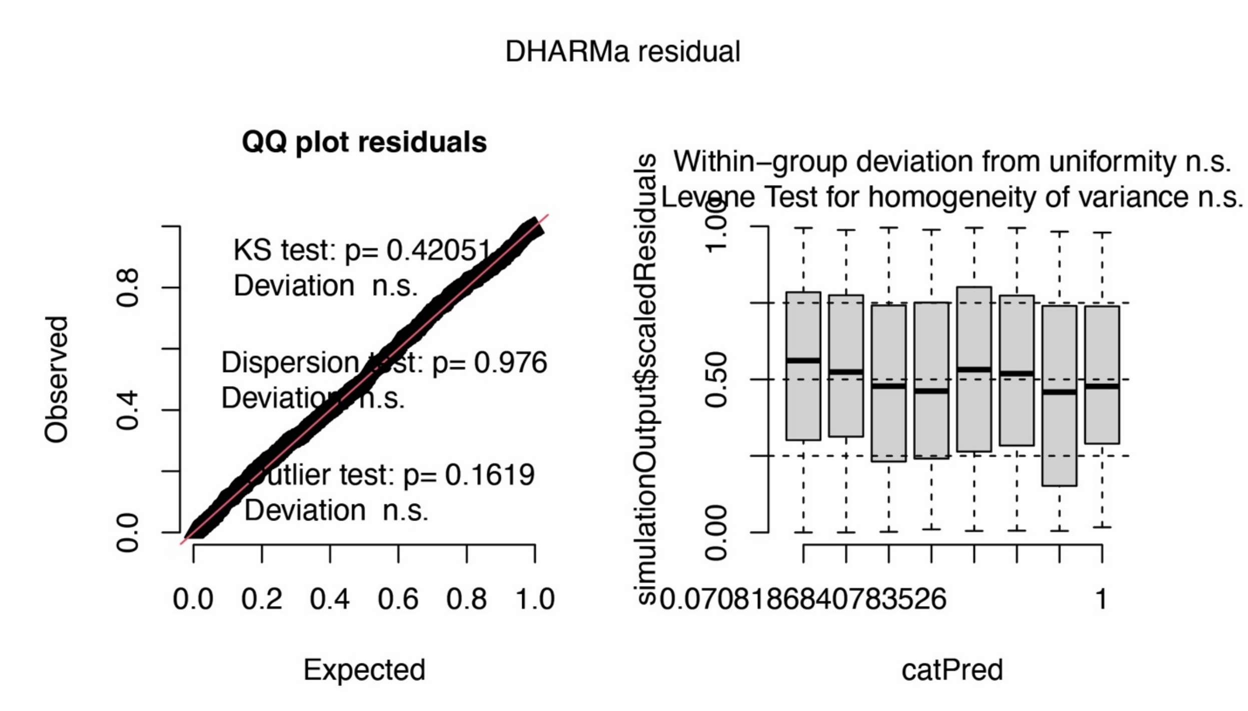

Figure S5. DHARMa residual for the great hammerhead, showing that the binomial model appropriately represented the data.

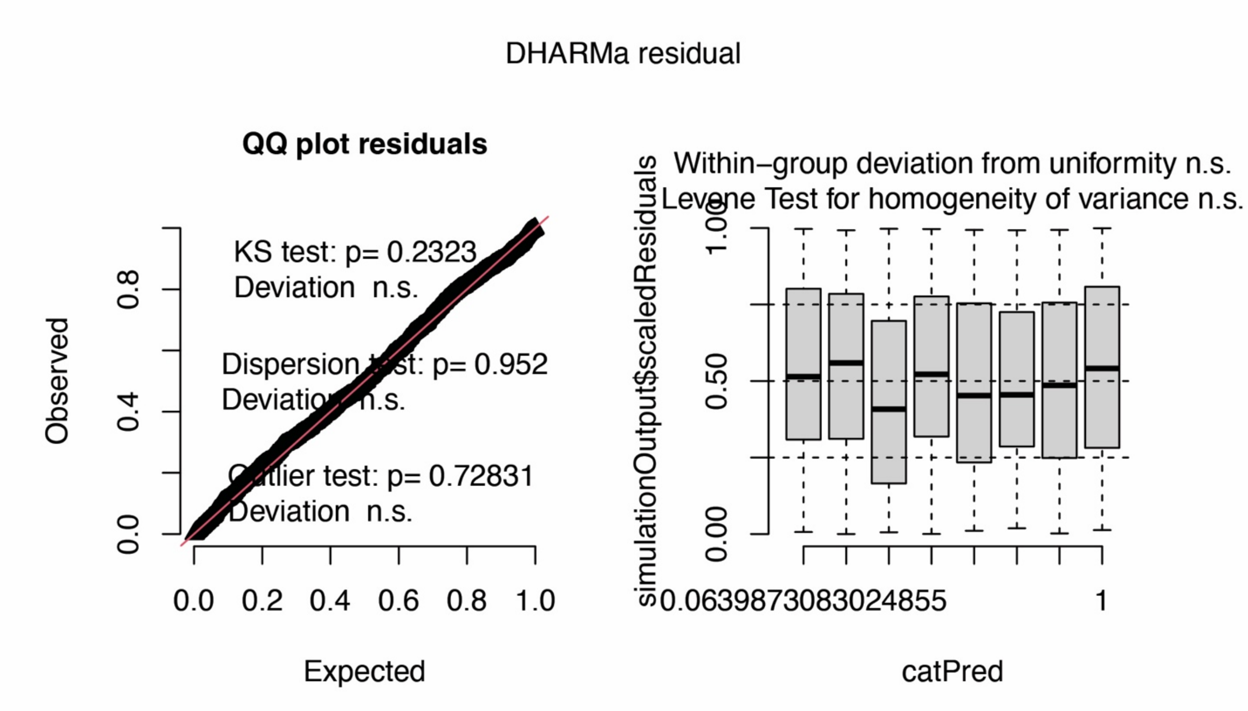

Figure S6. DHARMa residual for the oceanic whitetip shark, showing that the binomial model appropriately represented the data.

Table S1. AMOVA results for mtCR data for alternative grouping samples.

Groups	ϕ_{ct}	ϕ_{sc}	ϕ_{st}
Ant within Car	0.6041	**0.8205**	**0.5465**
Ant outside Car	**0.7007**	**0.8470**	**0.4887**

Significant values in bold ($p<0.05$).

Table S2. Population differentiation based on F_{ST} values between pairwise populations

	EPA	CPA	WPA	NWA	CAR	SWA	EAT	IOC	ANT
EPA	—								
CPA	**0.3155**	—							
WPA	**0.3496**	**0.2278**	—						
NWA	**0.9495**	**0.9763**	**0.6968**	—					
CAR	**0.9332**	**0.9497**	**0.6095**	**0.7313**	—				
SWA	**0.9572**	**0.9896**	**0.7115**	**0.8718**	**0.6149**	—			
EAT	**0.9456**	**0.9886**	**0.5783**	**0.8717**	**0.4643**	**0.9213**	—		
IOC	**0.9135**	**0.9202**	**0.5683**	**0.6829**	**0.6651**	**0.8351**	**0.6887**	—	
ANT	**0.9531**	**0.9905**	**0.6341**	**0.7049**	**0.7652**	**0.9650**	**0.9541**	**0.5659**	—

Significant values in bold ($p<0.001$)

Data S1.

Data S1 (.csv) with all reported trade for shark fins to CITES from 2015 to 2021 for commercial purposes, with specified volumes, for wild purposes, all exporting countries, Hong Kong as importer, for all CoP16 shark species.

Data S2.

DataS1 (.xlsx) with haplotype frequencies from population sources and mixed populations from the fin trimming and small fin survey

Data S3.

DataS2 (.xlsx) with Hong Kong market species composition and counts of shark fin trimmings per species per year.
